# Olanzapine Reverses MK-801-Induced Cognitive Deficits and Region-Specific Alterations of NMDA Receptor Subunits

**DOI:** 10.3389/fnbeh.2017.00260

**Published:** 2018-01-09

**Authors:** Xiao Liu, Jitao Li, Chunmei Guo, Hongli Wang, Yaxin Sun, Han Wang, Yun-Ai Su, Keqing Li, Tianmei Si

**Affiliations:** ^1^Institute of Psychology, North China University of Science and Technology, Tangshan, China; ^2^The Sixth People’s Hospital of Hebei Province, Baoding, China; ^3^National Clinical Research Center for Mental Disorders (Peking University Sixth Hospital/Institute of Mental Health) and The Key Laboratory of Mental Health, Ministry of Health (Peking University), Beijing, China

**Keywords:** MK-801, olanzapine, cognition, schizophrenia, NMDA receptor, prefrontal cortex

## Abstract

Cognitive dysfunction constitutes an essential component in schizophrenia for its early presence in the pathophysiology of the disease and close relatedness to life quality of patients. To develop effective treatment of cognitive deficits, it is important to understand their neurobiological causes and to identify potential therapeutic targets. In this study, adopting repeated MK-801 treatment as an animal model of schizophrenia, we investigated whether antipsychotic drugs, olanzapine and haloperidol, can reverse MK-801-induced cognitive deficits and how the reversal processes recruited proteins involved in glutamate neurotransmission in rat medial prefrontal cortex (mPFC) and hippocampus. We found that low-dose chronic MK-801 treatment impaired object-in-context recognition memory and reversal learning in the Morris water maze, leaving reference memory relatively unaffected, and that these cognitive deficits can be partially reversed by olanzapine, not haloperidol, treatment. At the molecular level, chronic MK-801 treatment resulted in the reduction of multiple N-methyl-D-aspartate (NMDA) receptor subunits in rat mPFC and olanzapine, not haloperidol, treatment restored the levels of GluN1 and phosphorylated GluN2B in this region. Taken together, MK-801-induced cognitive deficits may be associated with region-specific changes in NMDA receptor subunits and the reversal of specific NMDA receptor subunits may underlie the cognition-enhancing effects of olanzapine.

## Introduction

Schizophrenia is a severe, chronic psychiatric disorder that represents a major burden for patients, their families and the whole society (Howes and Murray, 2014). The disease is characterized by psychotic, negative and cognitive symptoms. Cognitive dysfunction is of particular importance, because cognitive improvement is closely related to prognosis and life quality in schizophrenic patients (Green, 2007; Matsui et al., 2008). Moreover, although psychotic symptoms such as hallucinations and delusions can be effectively relieved by existing antipsychotic drugs, cognitive deficits could be rescued by only some of second-generation antipsychotic drugs (SGAs, e.g., olanzapine), but not first-generation antipsychotic drugs (FGAs, e.g., haloperidol; Keefe et al., 2007; but see Dunlop and Brandon, 2015). To enhance the treatment effectiveness, it is important to understand the molecular mechanisms of schizophrenia-related cognitive deficits and to identify potential therapeutic targets.

Chronic treatment with the N-methyl-D-aspartate (NMDA) receptor antagonists, such as phencyclidine (PCP) and MK-801, produces wide-range schizophrenia-like behavioral, structural and neurobiological alterations and is thus a widely used animal model for schizophrenia (Beraki et al., 2008; Elsworth et al., 2011; Song et al., 2016). Adopting this model, several studies demonstrated the superiority of SGAs over FGAs in reversing cognitive deficits in spatial learning, reversal learning and recognition memory induced by NMDA receptor blockade (Abdul-Monim et al., 2006; Amitai et al., 2007; Grayson et al., 2007; Beraki et al., 2008; Goetghebeur and Dias, 2009; Song et al., 2016). These behavioral effects may be associated with differential neurobiological mechanisms of the two types of drugs in various aspects (Lieberman et al., 2008), such as the ability to block excessive cortical 5-HT efflux (López-Gil et al., 2007) and to prevent decreased neurogenesis following repeated NMDA receptor blockade (Maeda et al., 2007; Song et al., 2016). With regard to glutamate neurotransmission, previous studies found that SGAs such as olanzapine or clozapine, but not haloperidol, could prevent subchronic PCP-induced electrophysiological alterations of NMDA receptors *in vitro* (Ninan et al., 2003). The effects of these antipsychotic drugs on the expression of NMDA receptor subunits as well as proteins associated with glutamate release and clearance *in vivo* remain largely unknown.

In this study, we sought to examine the effects of olanzapine and haloperidol treatment on repeated MK-801-induced cognitive deficits and alterations in the expression levels of proteins involved in glutamate neurotransmission in rat medial prefrontal cortex (mPFC) and hippocampus. Cognition-related behaviors were measured in the object-in-context recognition task, reference memory and reversal learning in the Morris water maze. Functional NMDA receptors are comprised of two obligatory GluN1 subunits together with two GluN2 subunits of the same or different subtypes (Traynelis et al., 2010). Evidence from acute treatment of NMDA receptor antagonists indicates differential involvement of NMDA receptor subunits in NMDA receptor blockade-induced behavioral and prefrontal alterations (Jiménez-Sánchez et al., 2014). Here we assessed expression levels of NMDA receptor subunits in rat mPFC and hippocampus, including GluN1, GluN2A, GluN2B and their phosphorylated forms. Also evaluated were other proteins related to glutamate neurotransmission, including GluA1, a subunit of AMPA receptors, proteins involved in glutamate release (SNAP25, Syntaxin1 and VAMP) and clearance (excitatory amino acid transporter 2, EAAT2). Molecules associated with synaptic plasticity (PSD-95, glutamic acid decarboxylase-67 (GAD67), Nectin1 and Nectin3) were also examined.

## Materials and Methods

### Animals

Adult, male Sprague-Dawley (SD) rats (*n* = 44, age 9–10 weeks, weighing 300–350 g) were used in the present study. Purchased from the Laboratory Animal Science, Peking University Health Science Center, animals were housed four per cage in a controlled environment (23 ± 1°C; 45%–55% relative humidity; fixed 12/12 h light/dark cycle, lights on at 08:00 h) with food and water *ad libitum*. The study was carried out in accordance with the National Institute of Health’s Guide for the Use and Care of Laboratory Animals, the Peking University Committee on Animal Care and Use. The protocol was approved by the Peking University Committee on Animal Care and Use.

### Drug Treatment and Experimental Design

MK-801 (dizocilpine, St. Louis, MO, USA) was dissolved in normal saline. Haloperidol (Ortho-McNeil Pharmaceutical Inc., Titusville, NJ, USA) and olanzapine (Eli Lilly and Company, Indianapolis, IN, USA) were acidified by several drops of acetic acid and dissolved in normal saline.

**Figure 1:**
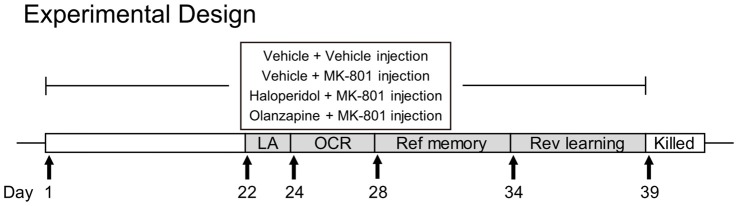
The experimental timeline of drug administration and behavioral tests. LA, locomotor activity; OCR, object-in-context recognition; Ref, reference; Rev, reversal.

Drug treatment started 7 days after animals’ arrival at our laboratory. Animals were randomly assigned into four groups (*n* = 11 per group): the vehicle + vehicle group (Veh), the vehicle + MK-801 group (MK), the haloperidol + MK-801 group (HPD), and the olanzapine + MK-801 group (Ola). For each group, animals received two intraperitoneal injections (with an interval of 30 min) daily between 15:00 h and 17:00 h for 3 weeks, with the first injection being vehicle or antipsychotic drugs and the second being vehicle or MK-801. At the 22nd day of drug injection, a series of behavioral tests were performed, including locomotor activity, object-in-context recognition, reference memory and reversal learning tests in the Morris water maze. Throughout the course of behavioral testing, drug treatment was continued. The doses of MK-801, haloperidol and olanzapine were 0.1 mg/kg, 0.02 mg/kg, and 0.5 mg/kg, respectively. Some randomly-chosen animals (*n* = 6 per group) were sacrificed in the next morning after the last drug treatment for western blot analyses. The timeline of drug treatment and behavioral tests is shown in Figure 1.

### Behavioral Tests

*Locomotor activity* was measured by an automated video tracking system (Shanghai Jiliang Software Technology Co., China) within a black soundproof chamber (40 cm × 40 cm × 65 cm) equipped with 5-W lamps in sidewalls and an overhead video recorder. Animals were individually placed in the chamber and allowed to freely explore it for 60 min. The locomotor activity was calculated as the total distance traveled in the chamber during 60 min using the DigBehv software.

*The object-in-context recognition task* was used to examine the memory of associating objects with their surrounding contexts (Balderas et al., 2008). Two black boxes (Context 1: 40 cm × 40 cm × 65 cm; Context 2: 60 cm × 60 cm × 50 cm) served as contexts. Multiple colored paper patches were attached to the two boxes to facilitate the discrimination of the two boxes. Objects included cones (Object A) and pyramids (Object B). Following Balderas et al. (2008) with minor modifications, the task consisted of two sample sessions and one test session. In the first sample session, animals were placed in the Context 1 and freely explored two identical Objects A for 10 min to learn the association between Object A and Context 1. Sixty minutes later, animals were introduced into the Context 2 to explore two identical Objects B for another 10 min. After a certain delay period (short-term, 60 min; long-term, 24 h) came the test session, during which animals were re-introduced into with either context with both an Object A and an Object B inside for 5 min. A preference index for new association between the object and its context (Object A in Context 2 or Object B in Context 1) was calculated as the amount of time exploring the spatially novel object divided by total time exploring both objects in the test session.

*Reference memory* and* reversal learning* were tested in the Morris water maze using established protocols (Vorhees and Williams, 2006). Experiments were performed in a circular pool (185 cm in diameter, 45 cm in height, made from dark plastic) filled with tap water (thermostatically controlled at 22 ± 1°C), with a circular platform (9 cm in diameter). Four cued trials were performed in the day before reference memory testing, in which a visible platform was placed in the center of the maze, 2 cm above the water’s surface, and animals were placed individually in the pool facing the wall. After all the trials, animals could successfully climb onto the platform, ruling out apparent visual and motor problems. The reference memory task consisted of five consecutive days of training to acquire the location of the platform, submerged 1 cm below the water surface, followed by a probe test on the sixth day. The pool was divided into four equal imaginary quadrants (I, II, III and VI) and the platform was located at the center of Quadrant I. On each training day, rats received four swimming trials with each starting from different locations. Animals finding the platform within 60 s were allowed to sit on it for 15 s and those failing to do so were guided by the experimenter to the platform and allowed to sit on it for 15 s. The inter-trial interval was 20 s. After training, animals were dried with a towel and put in a clean cage to avoid interaction with other animals. In the probe test, rats were placed in the pool without the platform to swim for 60 s. The day after the probe test came the reversal learning task, which was performed using the similar procedure with the reference memory task, except that the platform was moved to the center of the opposite quadrant (from Quadrant I to Quadrant III) and that animals were trained for four consecutive days followed by a probe test on the fifth day. The swimming performance of animals were recorded by a camera suspended above the pool center and analyzed using a video tracking and analysis system. Water maze performance in the acquisition phase was expressed as the mean escape latency of four trials in a training day. For the probe tests, the percentage time animals swam in the target, adjacent and opposite quadrants were used as an indication of spatial memory.

### Western Blot

Using Western blot, we examined the protein levels of NMDA receptor subunits (GluN1, GluN2A, GluN2B and their phosphorylated forms), AMPA receptor subunit GluA1 and other molecules (SNAP-25, Syntaxin1, VAMP, EAAT2, PSD-95, glutamic acid decarboxylase-67 (GAD67), Nectin1 and Nectin3). Rats were deeply anesthetized with pentobarbital (40 mg/kg of body weight, i.p.) and their brains rapidly removed and dissected to obtain mPFC and the whole hippocampus. Tissue from individual rats was immediately homogenized on ice in ice-cold lysis buffer (137 mM NaCl, 20 mM Tris–HCl (pH 8.0), 1% NP-40, 10% glycerol, 1 mM PMSF, 10 mg/ml aprotinin, 1 mg/ml leupeptin, 0.5 mM sodium vanadate), sonicated and centrifuged. The supernatants were stored at −80°C until required.

Samples containing 20 μg of protein were resolved by 10% acrylamide gels using Laemmli–SDS-PAGE, and transferred electrophoretically to a polyvinylidene difluoride (PVDF) membrane (Millipore, Bedford, MA, USA). The PVDF membranes (the pore size: 0.45 μm) containing the proteins of interest were then blocked with 5% non-fat milk diluted in Tris-buffered saline Tween (TBST) (150 mM NaCl, 10 mM Tris-HCl (pH 7.5) and 0.1% Tween) for 1 h at room temperature and incubated overnight at 4°C in primary antibodies diluted in TBST containing 5% non-fat milk (GluN1: rabbit anti-GluN1, 1:5000, ab-109182, Abcam, UK; phosphorylated GluN1: rabbit anti-pGluN1, 1:5000, 3381S, Cell Signaling, Danvers, MA, USA; GluN2A: rabbit anti-GluN2A, 1:5000, 4205S, Cell Signaling; phosphorylated GluN2A: rabbit anti-pGluN2A, 1:5000, ab-16646, Abcam; GluN2B: rabbit anti-GluN2B, 1:10,000, 135302, Synaptic Systems, Germany; phosphorylated GluN2B: rabbit anti-pGluN2B, 1:2000, ab-81271, Abcam; GluA1: mouse anti-GluA1, 1:1000, 182011, Synaptic Systems; PSD-95: mouse anti-PSD-95, 1:1000, SC-32290, Santa Cruz Biotechnology, Santa Cruz, CA USA; SNAP25: mouse anti-SNAP25, 1:10,000, 111011, Synaptic Systems; Syntaxin1: mouse anti-Syntaxin1, 1:5000, 110011, Synaptic Systems; VAMP1: rabbit anti-VAMP1, 1:10,000, 104,002, Synaptic Systems; EAAT2: rabbit anti-EAAT2, 1:10,000, ab-41621, Abcam; GAD-65/67: rabbit anti-GAD65/67, 1:5000, ab-49832, Abcam; Nectin1: rabbit anti-Nectin1, 1:2000, SC-28639, Santa Cruz Biotechnology; Nectin3: rabbit anti-Nectin1, 1:5000, SC-28637, Santa Cruz Biotechnology; β-actin: mouse anti-β-actin, 1:40,000, 3700S, Cell Signaling). The next day, membranes were rinsed three times with TBST (8 min each time) and incubated for 2 h with horseradish peroxidase-conjugated goat anti-rabbit or anti-mouse secondary antibodies (1:2500–20,000, Zhongshan Gold Bridge Biotechnology Corporation, China) diluted in TBST. Following another three TBST rinses, proteins of interest were visualized using an ECL system (Pierce, Rockford, IL, USA) and Kodak XBT-1 film. For saturation detection, we plotted the intensity histogram for each band using the NIH ImageJ software and validated that pixel intensities were distributed over a range rather than saturating at one end. The immunoreactive signals of the target proteins were quantified by densitometry and the values were corrected based on their corresponding β-actin levels. All results were normalized by taking the value of the vehicle group as 100%.

### Statistical Analyses

All data were expressed as means ± SEM. Data points more than two standard deviations from the mean were considered as outliers and excluded from further analysis. The Shapiro-Wilk test was used for normality check. For normally distributed data, group differences in behavioral measures and protein levels were tested using one-way analysis of variance (ANOVA), followed by the Tukey’s *Post Hoc* test. Repeated measures ANOVA was performed for distance traveled in the locomotor activity test every 5 min, the escape latency across training days for water maze tests, with treatment as the between-group factor and time as the within-group factor. The percentage time animals swam in the target, adjacent and opposite quadrants in the probe sessions of water maze tests was compared with each other using the Wilcoxon signed rank test. For data that were not normally distributed, group differences were examined using the Kruskal-Wallis test, followed by Mann-Whitney U tests. The significance level for all statistical tests was *P* < 0.05; a trend to approach significance with *P* < 0.1 was also reported.

## Results

### MK-801-Induced Decrease in Locomotor Activity Was Not Restored by Either Olanzapine or Haloperidol

**Figure 2:**
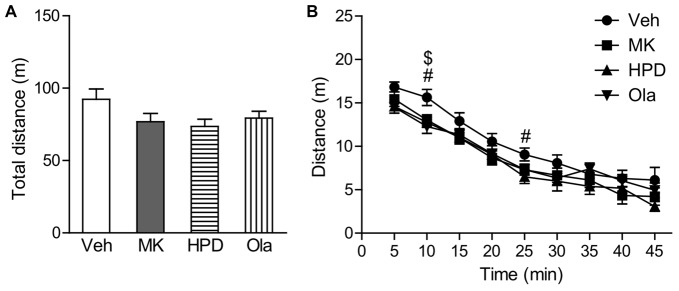
Effects of chronic MK-801 treatment and pretreatment with olanzapine or haloperidol on locomotor activity test. Total distance **(A)** and distance traveled every 5 min **(B)** in the locomotor activity test are presented. Values are expressed as means ± SEM (*n* = 11 per group). ^#^*p* < 0.05, Veh vs. HPD; ^$^*p* < 0.05, Veh vs. Ola. Veh, vehicle + vehicle; MK, vehicle + MK-801; HPD, haloperidol + MK-801; Ola, olanzapine + MK-801.

One-way ANOVA on total distance traveled in the locomotor activity test (Figure 2A) did not reveal significant group differences (*F*_(3,40)_ = 2.026, *P* = 0.126), although animals in the MK and HPD groups exhibited noticeable reductions in locomotor activity compared to vehicle animals. Analysis of distance traveled every 5 min (Figure 2B) using repeated measures ANOVA revealed a significant effect of time (*F*_(8,320)_ = 179.62, *P* < 0.001), without significant treatment effect (*F*_(3,40)_ = 2.026, *P* = 0.126) or interaction between treatment and time (*F*_(24,320)_ = 1.042, *P* = 0.412). Direct group comparisons for distance traveled every 5 min showed that compared to the vehicle group, animals in the HPD and Ola groups showed decreased locomotor activity in the first 10 min of the test (*P*s < 0.05). Similar reduction was noted in the MK group, but did not reach statistical significance.

### MK-801-Induced Deficits in Recognition Memory Were Partially Rescued by Olanzapine, Not Haloperidol

**Figure 3:**
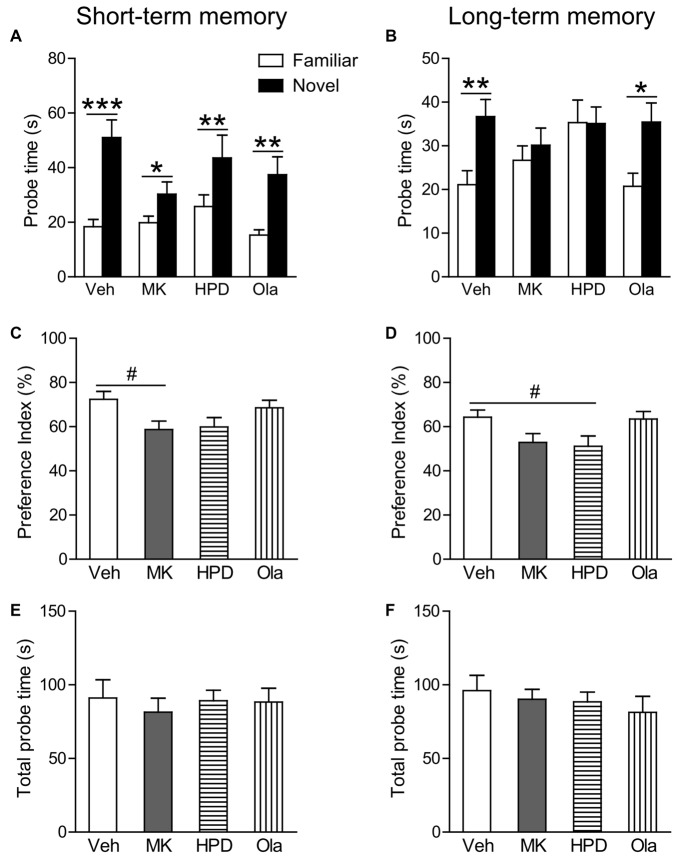
Olanzapine, but not haloperidol, partially prevented MK-801-induced deficits in short and long-term object-in-context recognition memory. **(A,B)** Mean probe time with the familiar or novel object in a certain context in the test phase. **(C,D)** The preference index (%) calculated as the amount of time exploring the novel object divided by total time exploring both objects in the test session. **(E,F)** Total probe time with the two identical objects in the acquisition phase. Values are expressed as means ± SEM (*n* = 10–11 per group). ^#^*p* < 0.1, *p < 0.05, **p < 0.01, ***p < 0.001. Veh, vehicle + vehicle; MK, vehicle + MK-801; HPD, haloperidol + MK-801; Ola, olanzapine + MK-801.

In the short-term probe session, all the groups spent significantly more time on the novel object than on the familiar object (*P*s < 0.044; Figure 3A), indicating that all groups successfully recognized the association between objects and their matched contexts. Further comparison of the preference index among groups (Figure 3C) using one-way ANOVA, however, revealed significant group differences (*F*_(3,38)_ = 3.031, *P* = 0.041), which was primarily driven by decreased preference index in the MK group compared to the Veh group, although this decrease did not reach statistical significance (*P* = 0.074). No group differences were observed for animals receiving haloperidol and olanzapine pre-treatment.

In the long-term probe session, only the vehicle and olanzapine groups distinguished the novel from the familiar object-context association by spending more time exploring the novel object in a given context (*P*s < 0.01; Figure 3B). Significant group differences were observed in the preference index (*F*_(3,39)_ = 3.169, *P* = 0.035), which was again driven by a noticeable reduction in the MK group compared to the Veh group (*P* = 0.065, Figure 3D). These results suggest that olanzapine pretreatment partially restored the ability of MK-801-treated animals to differentiate between the novel and familiar objects.

During sample sessions, no differences were observed among four groups in total time exploring both objects (Figures 3E,F).

### MK-801-Induced Reversal Learning Impairment Was Partially Rescued by Olanzapine, Not Haloperidol

**Figure 4:**
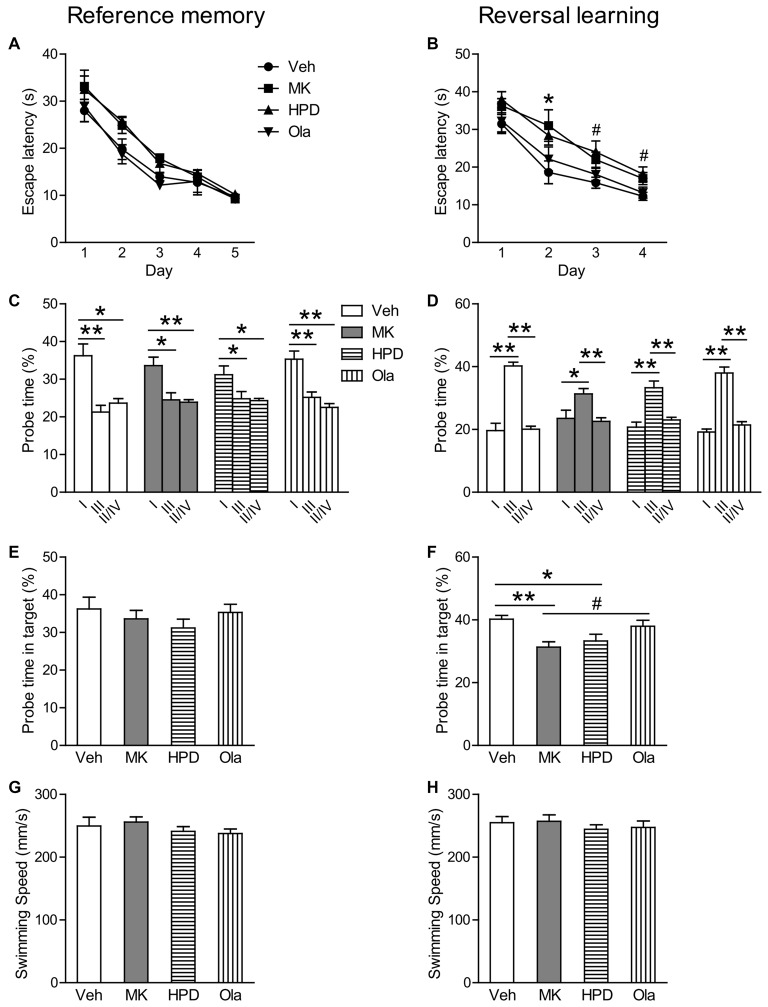
Olanzapine, but not haloperidol, reversed MK-801-induced reversal learning impairment in the Morris water maze test. **(A,B)** Mean escape latency in the reference memory and reversal learning tasks over training days. **(C,D)** The percentage time animals swam in the Quadrant I, III and II/IV in the probe tests. **(E,F)** The percentage time animals swam in the target in the probe tests. **(G,H)** Mean swim speed in the reference memory and reversal learning tasks over training days. Values are expressed as means ± SEM (*n* = 10–11 per group). For Panel **(B)**, *p < 0.05, Veh vs. MK; ^#^*p* < 0.05, Veh vs. HPD; for Panels **(C,D,F)**, ^#^*p* < 0.1, *p < 0.05, **p < 0.01. Veh, vehicle + vehicle; MK, vehicle + MK-801; HPD, haloperidol + MK-801; Ola, olanzapine + MK-801.

In the reference memory test, as shown in Figure 4A, group comparisons of mean escape latency across training days revealed a significant effect of day (*F*_(4,156)_ = 73.844, *P* < 0.001) and treatment (*F*_(3,39)_ = 6.121, *P* = 0.002), without significant interaction between the two factors (*F*_(12,156)_ = 0.576, *P* = 0.859). Analysis of mean escape latency on each day showed significant group differences on the second (*F*_(3,39)_ = 3.931, *P* = 0.015) and the third (*F*_(3,39)_ = 4.369, *P* = 0.010) training day, which was primarily driven by significant differences between HPD and Ola groups (*P* = 0.042) and between MK and Ola groups (*P* = 0.013), respectively. Although animals in the MK and HPD groups took longer time to locate the platform than the Veh group, *post hoc* comparisons did not reveal any significant differences among these groups. In the following probe test, all the four groups spent significantly more time swimming in the target than other quadrants (Figure 4C, *P*s < 0.041) and no significant treatment effects were found for the percentage of the time spent in the target quadrant (Figure 4E).

In the reversal learning task, repeated measures ANOVA on the escape latency over 4 days of training showed significant main effects of day (*F*_(3,117)_ = 51.966, *P* < 0.001) and group (*F*_(3,39)_ = 7.725, *P* < 0.001), without significant interaction of the two factors (*F*_(9,117)_ = 0.474, *P* = 0.889; Figure 4B). Analysis of mean escape latency on each day revealed significant group differences in all but the first training days (*F*s > 3.031, *P* < 0.041), because animals in the MK and HPD groups spent longer time to locate the platform (Day 2, MK vs. Veh, *P* = 0.048; Day 3 and 4, HPD vs. Veh, *Ps* < 0.045). In the probe test, although all the groups spent more time in the target than other quadrants (*P*s < 0.026, Figure 4D), significant group differences in the time percentage in the target quadrant were observed (*F*_(3,39)_ = 5.569, *P* = 0.003, Figure 4F). *Post hoc* comparisons showed that both MK and HPD groups showed significantly lower time percentage in the target quadrants compared to the Veh group (*P*s < 0.035), and olanzapine treatment partially reversed the reduction in the MK group (*P* = 0.055).

Finally, comparable swimming speeds among groups over the two training sessions were observed (Figures 4G,H), suggesting that MK-801-induced reversal learning impairment was not due to motor activity deficits.

### MK-801-Induced Region-Specific Changes in NMDA Receptor Subunits Were Reversed by Olanzapine, Not Haloperidol

To uncover the molecular mechanisms underlying antipsychotic effects of MK-801-induced cognitive impairment, we investigated expression levels of proteins involved in glutamate neurotransmission in rat mPFC and hippocampus.

**Figure 5:**
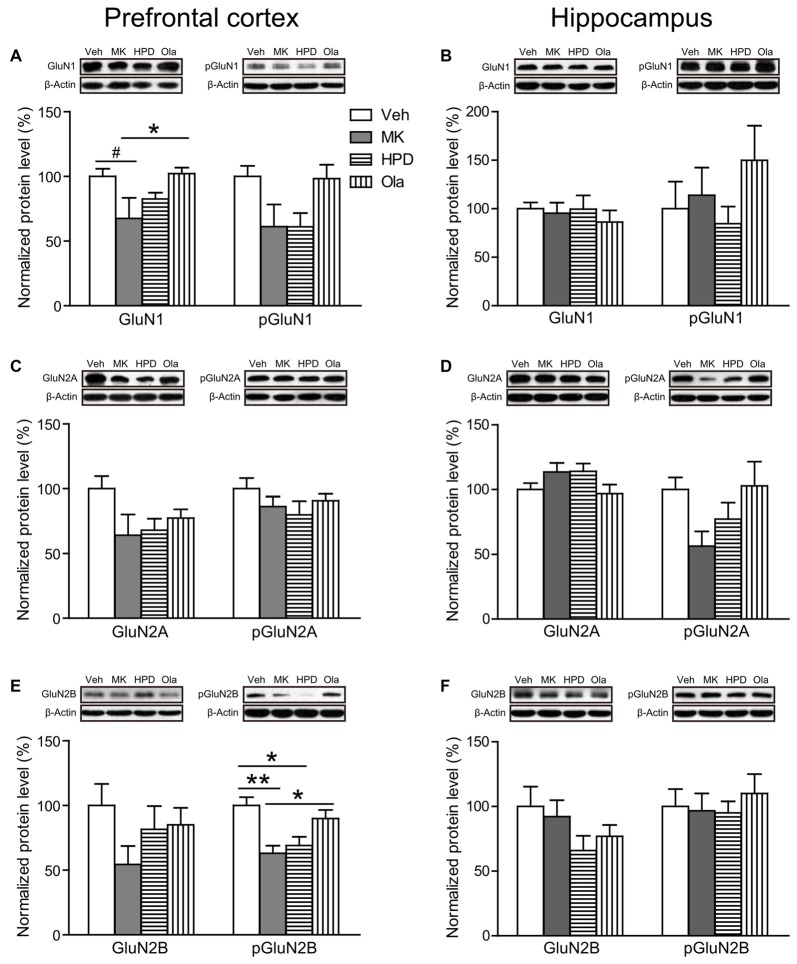
Effects of chronic MK-801 treatment and pretreatment with olanzapine or haloperidol on N-methyl-D-aspartate (NMDA) receptor subunits in rat medial prefrontal cortex (mPFC) and hippocampus. Representative Western blots and relative bar graphs showing the expression levels of **(A,B)** GluN1 and its phosphorylated form, **(C,D)** GluN2A and its phosphorylated form, **(E,F)** GluN2B and its phosphorylated form. Values are expressed as means ± SEM (*n* = 5–6 per group). ^#^*p* < 0.1, *p < 0.05, **p < 0.01. Veh, vehicle + vehicle; MK, vehicle + MK-801; HPD, haloperidol + MK-801; Ola, olanzapine + MK-801.

For NMDA receptor subunits (Figures 5A–F, compared to the Veh group, chronic MK-801 treatment caused widespread downregulation of NMDA receptor subunit expression in rat mPFC, which was noticeable in GluN1 (*P* = 0.068) and significant in phosphorylated GluN2B (*P* = 0.005). The reduction of phosphorylated GluN2B (*P* = 0.020) was also significant in the HPD group. Compared to the MK group, animals receiving olanzapine treatment exhibited significantly higher levels of GluN1 (*P* = 0.048), and phosphorylated GluN2B (*P* = 0.037), whereas haloperidol treatment had minimal influence on MK-801-induced reduction in NMDA receptor subunits (*P*s > 0.05). In rat hippocampus, the expression levels of NMDA receptor subunits in the hippocampus were comparable among all the groups.

**Figure 6:**
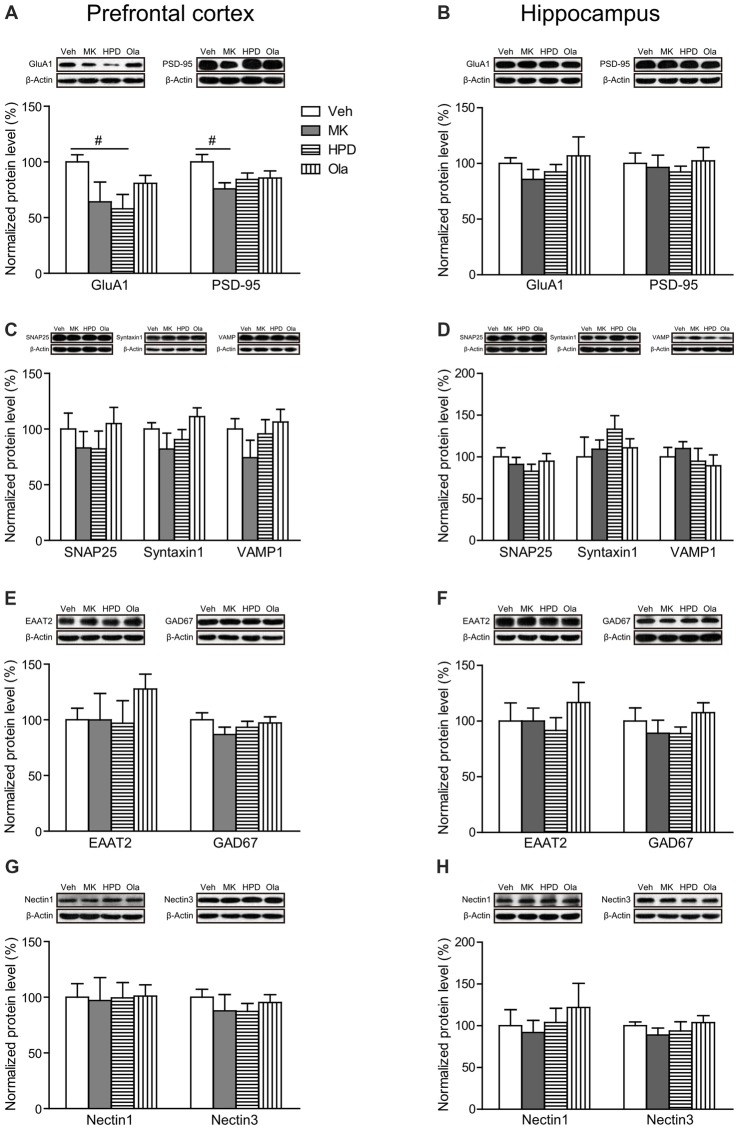
Effects of chronic MK-801 treatment and pretreatment with olanzapine or haloperidol on synaptic plasticity related molecules in rat mPFC and hippocampus. Representative Western blots and relative bar graphs showing the expression levels of **(A,B)** GluA1 and PSD-95, **(C,D)** SNAP25, Syntaxin1 and VAMP1, **(E,F)** EAAT2 and GAD67, **(G,H)** Nectin1 and Nectin3. Values are expressed as means ± SEM (*n* = 5–6 per group). ^#^*p* < 0.1. Veh, vehicle + vehicle; MK, vehicle + MK-801; HPD, haloperidol + MK-801; Ola, olanzapine + MK-801.

As shown in Figure 6A, there were trends for chronic MK-801 treatment to induce reduction in expression levels of GluA1 (a subunit of AMPA receptor, *P* = 0.070) and PSD-95 (*P* = 0.060) in rat mPFC. Neither olanzapine nor haloperidol was able to reverse these reductions. In comparison, these two molecules were unaffected by MK-801 in rat hippocampus (Figure 6B).

For molecules involved in presynaptic glutamate release (SNAP25, Syntaxin1 and VAMP1) and glutamate clearance (EAAT2), we did not observe any significant differences in their expression levels among all the groups in either mPFC or hippocampus (*P*s > 0.05, Figures 6C–F). The GABAergic neuron marker GAD67 also showed comparable expression levels among all the groups in both regions (*P*s > 0.05, Figures 6E,F).

Finally, recent evidence indicates that cell adhesion molecules, such as Nectin1/Nectin3, play essential roles in cognitive functions (Wang et al., 2013). Here we examined the influence of chronic MK-801 treatment on the expression levels of Nectin1 and Nectin3 in both mPFC and hippocampus and found that neither of them was significantly altered by chronic MK-801 treatment or the combination treatment of MK-801 and antipsychotic drugs (*P*s > 0.05, Figures 6G,H).

## Discussion

In this study, we examined the effects of olanzapine and haloperidol on MK-801-induced cognitive deficits and proteins involved in glutamate neurotransmission in rat mPFC and hippocampus. We found that chronic MK-801 treatment induced deficits in object-in-context recognition and reversal learning, leaving reference memory relatively unaffected and that these cognitive deficits can be (partially) reversed by olanzapine, not haloperidol, treatment. Moreover, such reversal effects may be associated with the upregulation of prefrontal GluN1 and GluN2B levels. Taken together, the cognition-enhancing effect of olanzapine may involve region-specific changes in NMDA receptor subunits.

Similar with our previous findings (Li et al., 2013), we found that chronic MK-801 treatment resulted in reduced locomotion in adult rats. As shown in the Figure 2B, MK-801-induced reduction in locomotor activity was manifest during the first 10 min of the test, when the animal activity is thought to be primarily driven by curiosity about new environment (Simpson et al., 2010). This result might be associated with amotivation caused by repeated MK-801 treatment (Paine and Carlezon, 2009) and future studies with multiple motivation measures are needed to validate this speculation. Interestingly, the decreased locomotor activity cannot explain cognitive deficits we observed, since in the cognitive tests, the measures reflecting basic motor abilities were comparable between vehicle and MK-801 groups such as total exploration time during the acquisition phase of recognition memory and swimming speed in the water maze. The differential MK-801 effects in the motor activity in different tasks could indicate an interaction between motor activity and task demands. Moreover, unlike cognitive deficits, the decreased locomotor activity in MK-801-treated animals was not reversed, but worsened, by antipsychotic treatment, which may be related to the different neural circuits supporting these behaviors.

The results that MK-801-induced cognitive deficits in recognition memory and reversal learning were rescued by olanzapine, but not haloperidol, are in agreement with previous findings that cognitive impairments induced by repeated NMDA receptor blockade can be reversed by SGAs, but not FGAs (Abdul-Monim et al., 2006; Grayson et al., 2007; Song et al., 2016). Our finding of preserved spatial learning performance in MK-801-treated animals does not completely disagree with the study of Song et al. (2016), which reported deficits in spatial learning, because both studies showed the longer, although not significant, escape latency for the MK-801-treated animals compared to animals in the Vehicle group in the first five training days. An inconsistency between the two studies is that as training days extended, the MK-801 effects diminished in our study, but became more pronounced in Song et al.’s (2016) study. This may be attributed to the time interval between MK-801 treatment and behavioral testing: MK-801 was administered about 20 h before behavioral testing in our study, but 20 min before behavioral testing in Song et al.’s (2016) study, in which acute effects of MK-801 treatment cannot be excluded.

We further investigated the role of glutamate neurotransmission in the cognition-enhancing effects of olanzapine. Our results showed that chronic MK-801 treatment resulted in region-specific changes in postsynaptic ionotropic glutamate receptors, with minimal influence on the expression levels of proteins involved in glutamate release and clearance. Specifically, the prefrontal cortex was associated with significant reduction in multiple subunits, whereas no significant changes were observed in the hippocampus. This finding accords well with the notion that these two regions are differentially affected by repeated NMDA receptor blockade (Jentsch and Roth, 1999; Lindahl and Keifer, 2004; Molteni et al., 2008; Kjaerby et al., 2017). It is worth noting that brain tissues were extracted from the entire hippocampus in this study. Considering that glutamate receptor subunits are differentially expressed and regulated in dorsal and ventral hippocampus (Pandis et al., 2006; Pacheco et al., 2017), future studies are needed to scrutinize MK-801-induced effects on glutamate receptor subunits in each subregion and to examine how these effects can be modulated by antipsychotic treatment.

The widespread reductions in NMDA receptor subunits, GluA1 (a subunit of AMPA receptors), and PSD-95 in the mPFC following repeated MK-801 treatment indicate abnormal glutamate neurotransmission in this region. Similar reduction of GluN1 and GluN2B subunit protein levels has been reported with chronic PCP treatment (Lindahl and Keifer, 2004). It has been reported that acute treatment of low-dose MK-801 preferentially decreases the activity of cortical inhibitory interneurons, resulting in a local disinhibition of pyramidal neurons (Homayoun and Moghaddam, 2007). This may be followed by an increase in extracellular glutamate, which persisted even after repeated NMDA receptor blockade (Amitai et al., 2012). The decreased expression levels of glutamate receptor subunits following repeated MK-801 treatment we observed here could be a compensatory response to elevated glutamate activity (Nakazawa et al., 2017).

Critically, olanzapine, but not haloperidol, partially reversed MK-801-induced reduction in NMDA receptor subunits. Given that olanzapine binds to various monoamine receptors including serotonin and *D*_2_ receptors, the superiority of olanzapine over the *D*_2_ antagonist haloperidol in restoring cognitive impairments may be attributed to the combined effect of blocking receptors not limited to *D*_2_ receptors (Lieberman et al., 2008). Particularly in the prefrontal cortex, olanzapine may function through the interaction among serotonin receptors, Ca^2+^/calmodulin-dependent protein kinase II (CaMKII) and the GluN2B subunit of the NMDA receptor (Purkayastha et al., 2012). Given the essential role of GluN2B in PFC-mediated cognitive functions (Monaca et al., 2015), the ability of olanzapine to reverse GluN2B expression levels in rat mPFC may contribute to its cognition-enhancing effects for MK-801-induced cognitive deficits. Finally, the failure of olanzapine to restore the reduction of GluA1, and PSD-95, together with previous observation that SGAs failed to modulate PCP-induced reductions in prefrontal metabolic activity (Cochran et al., 2003), suggests that the disrupted frontal functions caused by repeated NMDA receptor blockade cannot be fully restored by olanzapine treatment.

To conclude, the present study demonstrates that olanzapine, but not haloperidol, treatment partially reversed MK-801-induced cognitive deficits and region-specific alterations of NMDA receptor subunits. These results indicate the region-specific involvement of NMDA receptor subunits in the therapeutic effects of olanzapine and call for future studies to elucidate the interaction among multiple neurotransmitter systems and brain regions responsible for cognitive deficits in schizophrenia.

## Author Contributions

KL and TS designed the research. JL, XL, HoW, HW and YS performed the research. JL, XL, CG and Y-AS analyzed the data. JL, XL and TS wrote the manuscript.

## Conflict of Interest Statement

The authors declare that the research was conducted in the absence of any commercial or financial relationships that could be construed as a potential conflict of interest.
